# Quantifying Sexual Dimorphism by Analyzing Ramus Flexure and Bigonial Width in Orthopantomography

**DOI:** 10.7759/cureus.61848

**Published:** 2024-06-06

**Authors:** Abirami Arthanari, Shanmathy Sureshbabu, Karthikeyan Ramalingam, Vignesh Ravindran, Lavanya Prathap

**Affiliations:** 1 Department of Forensic Odontology, Saveetha Dental College and Hospitals, Saveetha Institute of Medical and Technical Sciences, Saveetha University, Chennai, IND; 2 Department of Oral Pathology and Microbiology, Saveetha Dental College and Hospitals, Saveetha Institute of Medical and Technical Sciences, Saveetha University, Chennai, IND; 3 Department of Pedodontics and Preventive Dentistry, Saveetha Dental College and Hospitals, Saveetha Institute of Medical and Technical Sciences, Saveetha University, Chennai, IND; 4 Department of Anatomy, Saveetha Medical College and Hospitals, Saveetha Institute of Medical and Technical Sciences, Saveetha University, Chennai, IND

**Keywords:** metric analysis, planmeca software, orthopantomogram, dimorphism in sex, anthropometric analysis, confidence interval, bigonial width, ramus flexure, mandibular parameters, sex analysis

## Abstract

Introduction

The mandible constitutes one of the skull's largest and strongest bones. Growth spurts can influence it, and it has a variety of dimorphic traits that can be used to identify sex. In addition to observing, comparing, and evaluating the potential for mandibular ramus flexure and bigonial breadth to discriminate between sexes using digital orthopantomograms (OPG), a retrospective study was conducted to examine the validity of this method for sex estimation in the Indian population.

Aim and objective

This study aims to quantify sexual dimorphism by analyzing two mandibular parameters, the ramus flexure and the bigonial width, using orthopantomography (OPG). The objective is to determine the accuracy of sex determination using the ramus flexure and bigonial width.

Materials and methods

A total of 500 OPG images (250 males and 250 females) were analyzed using the Planmeca software (Helsinki, Finland). The ramus flexure was measured as the angle formed between the tangent to the inferior border of the mandible and the tangent to the posterior border of the ramus. The bigonial width was measured as the distance between the left and right gonion points. A statistical analysis was performed to assess sexual dimorphism and determine the accuracy of sex determination using these parameters. The study employed descriptive statistics, such as means and standard deviations, and an independent t-test to determine the significance of the characteristics in relation to males and females.

Results

The mean bigonial width for females was 193.3068 mm (SD = 13.51669 mm) and for males was 217.6308 mm (SD = 10.87453 mm), with a statistically significant difference (p = 0.000). The 95% confidence interval for the difference in the bigonial width between males and females was between -49.97173 mm and -43.93787 mm. For the ramus flexure, the mean was 0.0000 for both males and females (SD = 0.00000), with a significant difference between males and females (p = 0.003). The 95% confidence interval for the difference in the ramus flexure between males and females was between -0.59543 and -0.12457.

Conclusion

The results indicated significant sexual dimorphism in both the ramus flexure and bigonial width. This study demonstrated that the ramus flexure and bigonial width, measured using orthopantomography (OPG), exhibited significant sexual dimorphism. The analysis of these mandibular parameters provided valuable information for sex determination in forensic and anthropological contexts.

## Introduction

The skull is perhaps the second most differentiated and easily sexed element of the skeleton after the pelvis, with an accuracy of up to 92%. When estimating gender in situations where a full skull is missing, the mandible can be highly significant. The mandible, the largest, strongest, and most dimorphic bone in the skull, is incredibly resilient and well-preserved because of its dense coating of compact bone. The mandibular ramus, in particular, can be utilized to differentiate between sexes, exhibiting strong univariate sexual dimorphism. Determining age and gender using mandibular characteristics is considered an essential component of forensic odontology and anthropology. Mandibular characteristics, including ramus length, height, and symphysis shape, might reveal a person's age and sex. Aging processes such as bone resorption can cause changes in the angle and form of the jaw, which can affect mandibular morphology and age estimation [1]. Mandibular traits such as strength and form that exhibit sexual dimorphism can help identify a person's sex. Forensic specialists can make a substantial contribution to the identification of human remains, supporting both criminal investigations and anthropological studies, by carefully examining these characteristics in conjunction with other bone features [2].

In dentistry, extra-oral digital dental imaging is progressively becoming standard practice. The ability to store and retrieve digital radiography images at any time has made digital radiography an indispensable tool in the field of forensic anthropology. Age and gender can be ascertained by means of a variety of radiographic images, such as digital imaging, lateral cephalograms, panoramic radiographs, lateral oblique X-rays, and advanced imaging technologies like cone beam CT (CBCT), MRI, and CT scans. The most often utilized extra-oral radiographs for assessing maxillo-mandibular structures in dentistry are orthopantomograms (OPGs) [3].

In forensics, ramus flexure analysis is a precise approach for determining age and sex. Age and sex have a noticeable impact on the angle that forms between the mandible's ascending and horizontal portions, known as the ramus flexure. The ramus flexure often decreases with age as a result of bone resorption and remodeling. Further, the ramus flexure is a clear indicator of sexual dimorphism, with males usually exhibiting a more obtuse angle than females. Forensic specialists can precisely determine age and sex by measuring and comparing ramus flexure angles; this allows them to assist in the identification of human remains and provide a scientific approach to forensic investigations [4].

In both odontology and anthropology, bigonial breadth is a valid marker of age and sex. The measurement of the distance between the mandibular gonion points is an anatomical metric that is associated with both sexual dimorphism and skeletal maturity. Bigonial width steadily grows during development until skeletal maturity is attained, at which point it largely stabilizes [5]. There is also obvious sexual dimorphism, with males usually having wider bigonial widths than females because of variations in bone strength. Forensic specialists may precisely determine age and sex by carefully examining bigonial width in conjunction with other bone data. This helps with both human identification and forensic investigations [6]. This study aims to quantify sexual dimorphism by analyzing two mandibular parameters, the ramus flexure and bigonial width, using OPG.

## Materials and methods

This study was employed to establish a population-specific criterion for evaluating sex and age with mandibular parameters as a measure. For this study, digital OPGs from the archives of the Department of Oral Medicine and Radiology at Saveetha Dental College and Hospital in Chennai were collected. The sample size was determined using G-Power software (version 3.1.9.4, Düsseldorf, Germany) to achieve a statistical power of 95% and a significance level (alpha error probability) of 0.05. The calculated sample size was 482, and we included a total of 500 samples to ensure adequate statistical power.

In this study, 500 OPGs of patients within the age group of 41 to 50 years constituted the sample population. The parameters evaluated were the ramus flexure and bigonial width. These parameters were assessed to determine whether they could contribute to age and sex determination. The OPGs were procured from the Planmeca Promax Scara 3 Digital OPG Machine (70 kVp, 8 mA for 09 seconds), manufactured by Planmeca (Helsinki, Finland) with a 1:1 ratio. The OPGs were transferred to Planmeca Romexis Viewer Software 2.9.2.R., and the measurements were recorded. The data were compiled in Microsoft Office Excel 2016 (Microsoft Corporation, Redmond, Washington). The statistical analysis was executed using IBM SPSS Statistics for Windows, Version 20 (Released 2011; IBM Corp., Armonk, New York). The OPGs with better clarity were included in the study. Ethical clearance was secured from Saveetha Dental College and Hospital Ethical Committee (IHEC/SDC/FACULTY/22/FO/059) before conducting the study. OPGs were categorized based on the inclusion and exclusion criteria. The digital tracing of the parameters for the included radiographs was performed using the Planmeca Romexis Viewer 2.9.2.R software.

Inclusion criteria

OPGs with proper radiographic patient positioning and mandibles with completely dentulous dentition were included in the study.

Exclusion criteria

The study excluded radiographs that showed signs of gross deformity of the maxilla-mandibular structures, any artifacts on the radiograph, radiolucent or radiopaque lesions in the mandibular arch, missing premolars, mixed dentition, history of trauma and/or ongoing or completed trauma therapy, and radiographic indications of temporomandibular joint (TMJ) issues.

Ramus flexure

**Figure 1:**
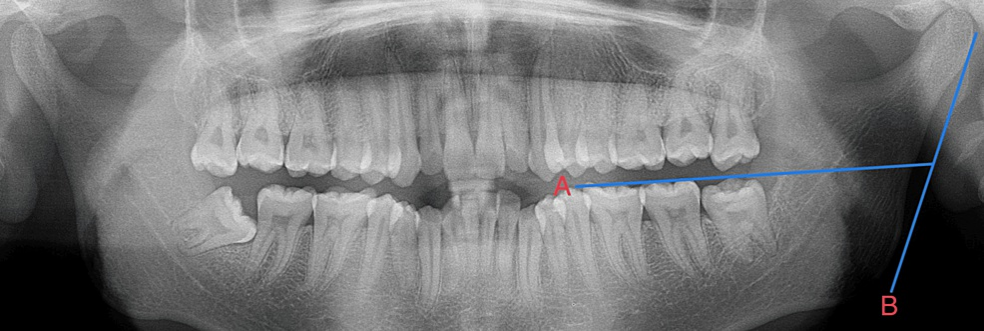
Ramus flexure at the occlusal plane (A) and the posterior border (B) seen in males

The ramus flexure is a distinct angulation present at the posterior border of the mandibular ramus, and the degree of ramus flexure in adult males can be observed at the occlusal surface of the molars, as illustrated in Figure 1.

**Figure 2:**
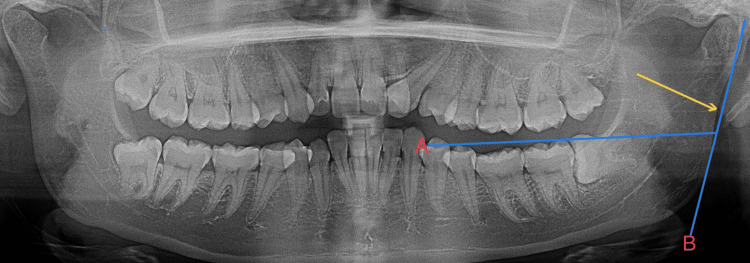
Ramus flexure present in the neck of the condyle (yellow arrow) seen in females

This method of observation has been obtained from Loth and Henneberg (1996) [7]. Following the image calibration (to obtain 1:1 magnification), the ramus flexure is traced on the OPG with two reference lines. One line is a tangent touching the posterior border of the ramus and the other line is a plane that connects the occlusal tips of the molars. In female subjects, the posterior border of the ramus can be straight, as shown in Figure 2.

**Figure 3:**
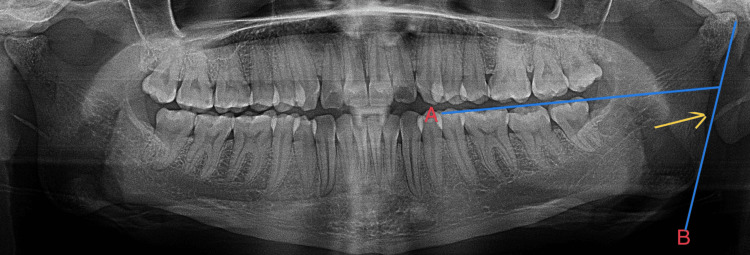
Ramus flexure below the occlusal plane (yellow arrow) seen in females

In female subjects, if the ramus flexure is observed, it is seen closer to the neck of the condyle, as shown in Figure 3.

**Figure 4:**
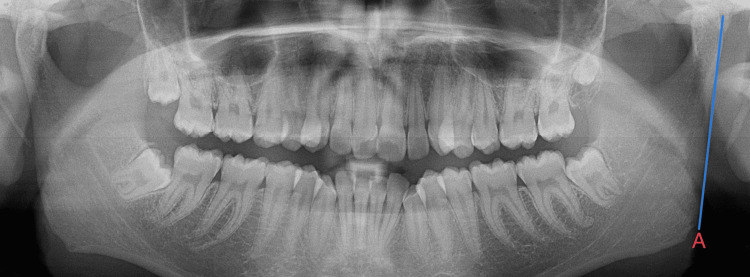
Ramus flexure absent (in blue) seen in females

In female subjects, the ramus flexure is observed below the occlusal plane in cases with gonial prominence or eversion, which are shown in Figure 4.

Bigonial width

**Figure 5:**
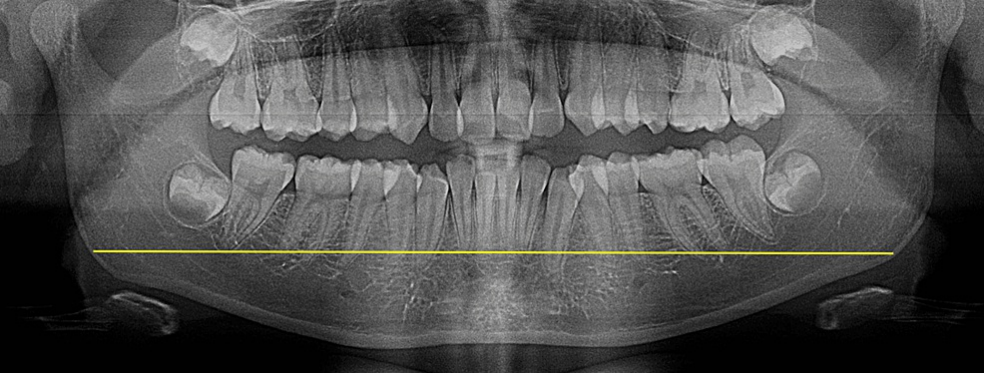
The measurement of the bigonial width

The bigonial width is the distance lapsed between the right and left gonia and is measured by calculating the horizontal area between the right and left gonia. The gonion (Go) lies in the external angle of the mandible, which is seen in the most inferior, posterior, and lateral points, as illustrated in Figure 5.

## Results

This study was undertaken to estimate age and determine sex using the mandibular ramus flexure and bigonial width. As compared and analyzed with the previous national and international studies, we restricted ourselves to descriptive statistics, where the mean and standard deviation were calculated. The data were subjected to an independent t-test, and the P-value was determined to establish the significance of the parameters for males and females. The mean bigonial width for females is 193.3068 mm, with a standard deviation of 13.51669 mm. The mean bigonial width for males is 217.6308 mm, with a standard deviation of 10.87453 mm. The P-value is 0.000, which is statistically significant and denotes a difference in the bigonial width between males and females. The 95% confidence interval for the bigonial width among males and females is between -49.97173 mm and -43.93787 mm. For the ramus flexure, the mean ramus flexure for both males and females is 0.0000, with a standard deviation of 0.00000. The P-value is 0.003, which is statistically significant and denotes a difference in the ramus flexure between males and females. The 95% confidence interval for the ramus flexure among males and females is between -0.59543 and -0.12457, as shown in Table 1.

**Table 1:** Analysis of “t” for the group statistics of 41 to 50 years The table shows the group statistics for two different measurements, bigonial width (mm) and ramus flexure, categorized by gender (male and female).

Group Statistics							95% Confidence Interval of the Difference	
	Gender	N	Mean	Std. deviation	Std. error mean	P-values	Lower	Upper
Bigonial width (mm)	Female	250	193.3068	13.51669	0.85487	0	-49.97173	-43.93787
	Male	250	217.6308	10.87453	0.68777	0	-49.97561	-43.93399
Ramus flexure	Female	250	0.00000	0.00000	0.00000	0.003	-0.59543	-0.12457
	Male	250	0.00000	0.00000	0.00000	0.003	-0.59543	-0.12457

The gender-based prediction of group membership yielded a classification rate of 92.4%, with 231 out of 250 male cases being correctly classified. For females, 215 out of 250 cases were correctly classified, which is 86.3%. Overall, 446 out of 500 cases were correctly classified, which is 89.4%. This means that the model correctly predicted the gender for 89.4% of the cases in the original group, as presented in Table 2.

**Table 2:** Prediction analysis of sex estimation using the ramus flexure and bigonial width The prediction analysis of the sex of males and females with the mandibular parameters shows 89.4% accuracy in predicting the correct sex.

GENDER	Predicted Sex		Total (N)
	Male (N)	Female (N)	
Male	231 (92.4%)	19 (7.6%)	250
Female	35 (13.7%)	215 (86.3%)	250
The overall percentage of 89.4% of the original group cases was correctly classified.			

## Discussion

Forensic odontology, also known as forensic dentistry, is a specialized field of dentistry that applies dental knowledge to legal investigations. It primarily involves the identification of human remains through dental records. In this study, a total of two mandibular parameters (in mm), namely, the ramus flexure and bigonial width, revealed statistically significant differences between the two genders. Therefore, the gender determination process helped develop a logistic regression model with different parameters that showed an accuracy of 89.4% in estimating gender, which may be applied in the field of forensic sciences.

Sex determination using morphometric characteristics such as the bigonial width and ramus flexure has been an intriguing aspect of forensic anthropology. These techniques explore the analysis of skeletal remains to determine a person's biological sex, offering insightful information for anthropological, archaeological, and forensic investigations. In the field of forensics, knowing how to use these skeletal traits to determine a person's gender advances our knowledge of human evolution, diversity, and population dynamics [8].

The angle that results from the intersection of the body of the mandible and the ramus is known as the ramus flexure. At birth, females have a more obtuse angle than males, for whom it usually displays a more acute angle [9]. It is believed that variations in hormonal impacts throughout growth and development are the cause of this sexual dimorphism. Because of the estrogenic effects on bone formation, females typically have a softer, more rounded angle, whereas males are more likely to have a sharper, more angular design because of the testosterone effect [10]. This angle must be carefully examined and quantified to assess and analyze the ramus flexure. This can be done using imaging methods such as CT scans, radiographic methods such as OPG, or by visual observation of the skeletal remains. Forensic anthropologists can determine the biological sex of a person under investigation by comparing the angle measured from the remains to standards set from known sex samples [11].

Similarly, the distance between the most lateral points on the angle of the mandible is referred to as the bigonial width. Sexual dimorphism of the bigonial width is characterized by wider angles in males than in females [12]. This difference results from variations in bone morphology caused by environmental, hormonal, and genetic causes. Male physiology is known to have larger muscle attachment sites, which are linked to higher total muscle mass and strength, and are responsible for the wider angles seen in males [13]. The process of measuring the bigonial width entails utilizing specialized anthropometric instruments or digital imaging software to precisely measure the distance between the mandibular angles [14]. After that, this measurement is correlated with reference ranges that have been defined for each sex and population group. Forensic anthropologists can determine whether an individual is a male or female based on the bigonial width, employing careful analysis and statistical validation [15].

Researchers and forensic experts can obtain information about an individual's biological sex by carefully examining these skeletal traits. This information can be used to resolve forensic cases, archaeological reconstructions, and anthropological studies [16]. Numerous studies and analyses conducted on a wide range of populations have demonstrated the statistical importance of the bigonial breadth and ramus flexure in determining sex. Research has repeatedly shown that there are unique patterns of sexual dimorphism, with females often displaying a smaller bigonial width and a more obtuse ramus angle than males [17]. These distinctions, which are linked to morphological changes and hormonal effects, are accurate markers of biological sex. Additionally, the discriminatory capacity of the bigonial width and ramus flexure has been strengthened by the application of statistical techniques that have improved their accuracy and reliability in sex determination evaluations [18]. It is critical to recognize the drawbacks and difficulties of determining a person's sex from their skeletal traits, though. The interpretation and reliability of sex evaluations can be affected by interpopulation variation, individual variability, and the influence of various factors. Despite these difficulties, forensic and biological anthropologists can make well-informed decisions about an individual's biological sex by combining ramus flexure and bigonial width analysis into a multifactorial approach. This helps us better understand human variation, evolution, and population dynamics [19].

Lin et al. in 2014 conducted a study on the Korean population in which they identified the sex of mandible models using their ramus flexure. All the variables of ramus flexure they employed in their study gave an accuracy of 88.8%. Hence, they concluded that the upper ramus above flexure is a valid tool in sex determination [4]. Research on the Indian population and their ability to show dimorphism in the ramus flexure was studied by Saini et al. in 2011. The authors identified that the ramus flexure gives an accuracy of up to 82%. They emphasized that in the Indian population, the ramus flexure may be used as a single dimorphic characteristic to determine sex [20]. Shah et al. (2020) studied mandibular dimensional changes in panoramic radiographs in which the bigonial width showed high statistical significance for sex. Thus, the authors deduced that the bigonial width can be best sought for gender determination [5]. Kano et al. in 2015 conducted research on the Japanese population by using post-mortem CT. The parameters in the study, along with the bigonial width, showed a statistical significance of P<0.001. With this, they concluded that the bigonial width can be preferred for sex determination among the Japanese adult population [14]. All of the abovementioned studies are in synchrony with the results achieved in this study. Both the parameters, ramus flexure and bigonial width, obtained statistical significance.

It is vital to recognize that the bigonial width and ramus flexure are not definite markers of biological sex, even though they do exhibit dimorphism traits. The precision and reliability of determining a person's sex using these skeletal traits can be affected by interpopulation variation, individual variability, and the influence of factors including age, nutrition, and pathology. Consequently, to improve the validity of sex determination, forensic anthropologists must utilize a multifactorial method that incorporates several skeletal indications and takes contextual information into account [21]. The application of the bigonial width and ramus flexure in sex determination is a useful technique in biological and forensic anthropology. These bone characteristics aid in forensic examinations, archaeological reconstructions, and anthropological research by offering important information about an individual's biological sex. To guarantee accuracy and dependability in sex determination analyses, their interpretation necessitates careful evaluation of numerous aspects and confirmation against established criteria [22].

Limitations

The limitation of this study is that the accuracy of sex determination using the ramus flexure and bigonial width may be influenced by interobserver variability. Different observers may interpret the OPG images differently, potentially affecting the accuracy of the measurements and sex determination. Additionally, the study may be limited by the sample size and demographic characteristics of the studied population, which could impact the generalizability of the results.

## Conclusions

This study demonstrates that the ramus flexure and bigonial width, measured using OPG, exhibit significant sexual dimorphism. The analysis of these mandibular parameters provides valuable information for sex determination in forensic and anthropological contexts. The high accuracy rate in sex determination, achieved by combining the ramus flexure and bigonial width, suggests the potential utility of OPG as a reliable method for sex determination. However, further studies with diverse populations are warranted to validate these findings and enhance the applicability of OPG in forensic and anthropological investigations.
